# Density distribution of gene expression profiles and evaluation of using maximal information coefficient to identify differentially expressed genes

**DOI:** 10.1371/journal.pone.0219551

**Published:** 2019-07-17

**Authors:** Han-Ming Liu, Dan Yang, Zhao-Fa Liu, Sheng-Zhou Hu, Shen-Hai Yan, Xian-Wen He

**Affiliations:** School of Mathematics and Computer Science, Gannan Normal University, Ganzhou, China; Chuo University, JAPAN

## Abstract

The hypothesis of data probability density distributions has many effects on the design of a new statistical method. Based on the analysis of a group of real gene expression profiles, this study reveal that the primary density distributions of the real profiles are normal/log-normal and t distributions, accounting for 80% and 19% respectively. According to these distributions, we generated a series of simulation data to make a more comprehensive assessment for a novel statistical method, maximal information coefficient (MIC). The results show that MIC is not only in the top tier in the overall performance of identifying differentially expressed genes, but also exhibits a better adaptability and an excellent noise immunity in comparison with the existing methods.

## 1 Introduction

A gene expression profile can indicate whether a particular gene has been expressed, expressed abundance, and the differentially expressed levels in different tissues, different development or physiological states. It plays an important role in studying the characteristics of an organism and its gene functions [1, 2]. A gene expression profile can also be used to identify differentially expressed genes (DEGs), helping to discover the biological processes and dysfunctions of the organism, and to understand the pathogenesis of diseases, the drug response and therapeutic effects. Gene expression analysis overcomes the shortcomings yielded from single gene analysis, and maximizes the integration of various biological information to comprehensively analyze the expression and functions of multiple genes during a disease development [3–6].

It is an important challenge of statistical methodology to identify differentially expressed genes from gene profiles [7]. In response to this challenge, many studies have proposed numbers of promising methods for gene expression analysis [5–28]. Maximal information coefficient (MIC) is a novel statistical method of data analysis [29]. We have successfully applied it to genome-wide association studies, identifying differentially expressed genes and miRNAs, and achieved good results [30–33]. In these studies, however, we just focused on the application of MIC, lacked fully evaluation MIC in terms of performance. In addition, in order to discover knowledge from a dataset, we often need to assume the probability density distribution of the dataset. The distribution hypothesis extremely affects the design of a new data mining method and the accuracy and interpretability of the results. So far a method used to analyze gene expression profiles usually (or potentially) assume that the probability density of the profiles is a normal distribution. Although the assumption is feasible in many cases, the feasibility may be not enough. And, there may be other distributions involved in gene expression data besides the normal.

This study attempts to explore the probability density distributions on real gene expression profiles. And, according to the real distributions, we generated a series of simulation datasets and employed SAM, Limma, ROTS and DESeq2 as benchmarks of MIC to illustrate its overall performance in identifying differentially expressed genes. Our experiments showed that the primary probability density distributions of real gene expression profiles are normal/log-normal distribution (~80%) and t-distribution (~19%) and a few Cauchy distributions (~1%). The simulation experiments on the four distributions reveal that MIC not only has the overall performance of identifying differentially expressed genes at the first tier comparing with the existing methods, but also its adaptability, especially the noise immunity are better than the existing methods, as well as its shorter runtime. Thus, MIC is an excellent method for identifying differentially expressed genes.

The first major contribution of this study is to explore the probability density distributions of real gene expression data, which might provide theoretical supports for an analysis of gene expression in the future, and overcome the lack of distribution hypothesis for that the hypothesis of probability distribution in current researches is usually just normal distribution. In addition to that the hypothesis of the probability distribution of a dataset might affect the design of a new method, existing methods have some limitations, at least partly [34]. Therefore, continuing to explore new methods for identifying differentially expressed genes is still an important task in bioinformatics. The second major contribution of the study is to comprehensively analyze the performance of the novel statistical method MIC in identifying differentially expressed genes on the real probability distributions, which could provide reference values for employing MIC to identify differentially expressed genes or other analysis.

## 2 Material and methods

### 2.1 Material

#### 2.1.1 Real gene expression profiles collection

All real profiles were obtained from the gene expression omnibus (GEO) on NCBI [35]. The data were randomly downloaded from GEO, by using the strategy ‘as many common species as possible’. There are totally 100 datasets with 20 species were collected in our study, shown in Table 1.

**Table 1:** Real gene expression profiles.

Sample	Count
Arabidopsis thaliana	6
Arachis hypogaea	1
Citrus limonia	1
Citrus reticulata	2
Citrus sinensis	7
Danio rerio	1
Drosophila melanogaster	6
Glycine max	5
Homo sapiens	32
Mus musculus	16
Oryctolagus cuniculus	1
Oryza sativa	4
Phaseolus coccineus	1
Rattus norvegicus	7
Solanum lycopersicum	1
Staphylococcus aureus	2
Staphylococcus aureus subsp. aureus str. Newman	1
Triticum aestivum	3
Triticum turgidum subsp. durum	1
Zea mays	2

#### 2.1.2 Simulation data

By the experiment of probability density analysis on real gene expression data in Section 3.1, we got four distributions, that is, normal, log-normal, t (Student) and Cauchy distribution. According to these distributions, a series of simulation datasets were generated.

To generate normal distribution simulation data, the parameters used in work [36] and [37] were employed. The parameters of three non-differentially and three differentially expressed genes of normal distribution were cross-combined into a total of 9 groups of parameters. And, the other three distribution parameters are listed in Tables 2–4, where are 16 groups totally. Each group of parameters was repeatedly generated into 100 datasets, each of which contains 6 cases and 6 controls, 10,000 genes (5% of which is 500 differentially expressed genes). In this way, a total of 25 groups, i.e. 2,500 datasets, were obtained. Among log-normal, t and Cauchy distributions, for each distribution, we generated one group of dataset with probability density curve shape significantly different from the real data, while the other groups is as close as possible to the real data.

**Table 2:** Parameters of log-normal distribution in simulation.

Group	Non-differential expression				Differential expression			
	Case		Control		Case		Control	
	*α*	*σ*	*α*	*σ*	*α*	*σ*	*α*	*σ*
1	5	1.5	5	1.5	4.5	1	5	2
2	5	1.5	5	1.5	5	1	6	1.1
3	5	1.5	5	1.5	6	0.8	6.5	1.2
4	5.5	1.3	5.5	1.3	4.5	1	5	2
5	5.5	1.3	5.5	1.3	5	1	6	1.1
6	5.5	1.3	5.5	1.3	6	0.8	6.5	1.2
7	7	1	7	1	4.5	1	5	2
8	7	1	7	1	5	1	6	1.1
9	7	1	7	1	6	0.8	6.5	1.2

**Table 3:** Parameters of t distribution in simulation.

Group	Non-differential expression				Differential expression			
	Case		Control		Case		Control	
	*df*	*ncp*	*df*	*ncp*	*df*	*ncp*	*df*	*ncp*
1	3	0	3	0	3	1	4	0
2	4	0	4	0	4	1	3	0
3	3	3	3	3	3	2	4	1
4	3	2	3	2	3	1	4	0
5	1.5	0	1.5	0	1.5	0	1.3	0.5

**Table 4:** Parameters of Cauchy distribution in simulation.

Group	Non-differential expression				Differential expression			
	Case		Control		Case		Control	
	*μ*	*λ*	*μ*	*λ*	*μ*	*λ*	*μ*	*λ*
1	1000	10	1000	10	1000	10	950	9
2	100	5	100	5	100	5	95	4

#### 2.1.3 Transformation of simulation data for DESeq2

DESeq2 is a method for RNA-seq data analysis. RNA-seq data is a discrete count dataset. Since a gene expression profile is continuous, it is necessary to convert the profile into discrete type. The conventional approach is to round off the expressed values into integers. The disadvantages of this approach include (1) a lot of information of the low expressed level genes will be lost; (2) the expressed values less than 0 cannot be processed; (3) the data with large variance (e.g., Cauchy distribution) may make DESeq2 fail. This study designed an algorithm (shown in Box 1) to make a transformation of the data to avoid the disadvantages.

**Box 1. Data transformation for DESeq2**

for *i* ← 1 to *cnt* /* *cnt* is the number of rows of dataset *d* */

    do if *cc* = 1 /* *cc* is a bool variable. *cc* = 1 represents Cauchy distribution,

            and *cc* = 0 is the other distributions. */

        then do if *d*[*i*] < -2*σ* /* *σ* is the standard deviation of *s* */

                then do remove *d*[*i*] /* remove the outlier */

                else *d*[i] ← *d*[*i*]*10

if test0(*d*) /* Function test0() is used to test there is any minus in *d*.

          It returns true if exists. */

  then do *d* + |min(*d*)| + 2 /* min(*d*) represents the minimum value in the dataset,

                        and +2 is used to reduce the count of ‘0’. */

for *i* ← 1 to *cnt*

  do round(*d*[*i*]) /* round off the values into integers */

Since a probability density curve is not deformed by scaling and translation, the algorithm will not affect the distribution of the data, that is, it will not affect the results of identifying differentially expressed genes of a method.

### 2.2 Methods

#### 2.2.1 Maximal information coefficient

Maximal information coefficient was proposed by David N. Reshef in 2011 to explore possible, undiscovered relationships between two variables [29]. It is a non-parametric statistical tool, thus, it can directly yield the degree of association between the two variables without assuming a mathematical model between the variables. So far, in a gene expression profile, there is no accepted mathematical model between sample phenotype and gene expressed value, therefore, MIC is a good choice for gene expression data analysis.

To calculate the MIC value, David N. Reshef et al. consider the bi-variable as points on a plane and divide the points into *x* and *y* bins in the horizontal and vertical axis. Thus, a grid with size of *xy* will be formed on the plane. MIC of dataset ***D*** with bi-variable can be defined as [29]

$$MIC\left(D\right)=\underset{xy<B(n)}{\max}\left\{M{\left(D\right)}_{x,y}\right\},$$ (1)

where, *n* represents the sample size, *B*(*n*) is the upper limit of the grid size (usually, *ω*(1)<*B*(*n*)<*O*(*n*^1−*ε*^), 0<*ε*<1), and ***M***(***D***) is the characteristic matrix of ***D***, which is defined by

$$M{\left(D\right)}_{x,y}=\frac{I^{*}(D,x,y)}{\log\;\min\{x,y\}},$$ (2)

I* is the mutual information between the two variables in ***D***.

The pair of features, 'sample phenotype' and 'gene expressed value' in a gene profile, can be considered as a bi-variable, so that the MIC value between the features can be calculated. Let the profile contains *N* samples, each sample having *L* genes, the phenotype *T* = (*t*_1_,*t*_2_,⋯,*t*_*N*_) ($t_{i}=\{\begin{array}{ll} 0, & controls\\ 1, & cases \end{array}$), the gene expressed vector *G* = (*g*_1_,*g*_2_,⋯,*g*_*L*_)^*T*^, where, *g*_*j*_ = (*g*_1*j*_,*g*_2*j*_,⋯,*g*_*Nj*_), *g*_*ij*_ represents the expressed value of the gene *j* in the *i*-th sample, then the mathematical model between gene *g*_*j*_ and its phenotype *T* can be simply described as a map

$$T=f(g_{j}).$$ (3)

Obviously, this is an abstraction that can represent any model. Therefore, regardless of the model of real gene expression profiles, the association between genes and disease can be inferred easily by MIC. The level of MIC value indicates the degree of an association between the gene and the disease.

#### 2.2.2 Benchmarks

For the purpose to evaluate the performance of MIC on identifying differentially expressed genes, four existing methods, DESeq2, Limma, ROTS and SAM were selected as the benchmarks in our experiments.

**1. DESeq2.** DESeq2 is an improved version of DESeq. DESeq performs analysis on massive RNA-seq data using a negative binomial (NB) model with mean and variance linked by local regression [20]. DESeq2 uses shrinkage estimators to achieve dispersion and fold change, reducing type I errors. After a necessary transformation, a gene expression data can be analysed by DESEQ2.

**2. Linear models for microarray.** Limma supposes that gene expression data meets a linear model [27]

$$E\left(y_{g}\right)=X\alpha_{g}$$ (4)

and

$$\mathrm{var}\;\left(y_{g}\right)=W_{g}\sigma_{g}^{2}$$ (5)

where ***y***_*g*_ is the expressed vector, ***X*** is a design matrix, ***a***_*g*_ is a coefficient vector, and ***w***_*g*_ is a known non-negative weight matrix.

Intergenic differences can be represented as

$$\beta_{g}=C^{T}\alpha_{g},$$ (6)

where ***C*** is the contrast matrix.

The linear model is fitted to the response variable to obtain an estimator $s_{g}^{2}$ of the coefficient estimators $\hat{\alpha_{g}}$ and $\sigma_{g}^{2}$. The contrast estimator is defined as $\hat{\beta_{g}}=C^{T}\hat{\alpha_{g}}$, and its covariance matrix estimator is

$$\mathrm{var}\;\left(\hat{\beta_{g}}\right)=C^{T}V_{g}Cs_{g}^{2},$$ (7)

where ***V***_*g*_ is an unscaled covariance matrix.

Limma's hypothesis of $\hat{\beta_{g}}$ and $s_{g}^{2}$ is to obtain a modified t-statistic

$$t_{gj}=\frac{\hat{\beta_{gj}}}{s_{g}\sqrt{v_{gj}}},$$ (8)

*v*_*gj*_ is the *j*-th diagonal element of *C*^*T*^*V*_*g*_*C*.

**3. Reproducibility-optimized test statistic.** Reproducibility-optimized test statistic (ROTS) performs well in microarrays, massive RNA-seq data and mass spectrometry-based proteomics data analysis [15, 28, 38].

ROTS maximizes the scaled reproducibility based on the parameter *α* = (*α*_1_, *α*_2_)(*α*_1_∈[0,∞), *α*_2_∈{0,1}) and the top list with size *k* [28],

$$\frac{R_{k}\left(d_{\alpha }\right)-R_{k}^{0}\left(d_{\alpha }\right)}{s_{k}\left(d_{\alpha }\right)},$$ (9)

where *s*_*k*_(*d*_*α*_) is the estimator of standard deviation of the bootstrap distribution of the observed reproducibility. *R*_*k*_(*d*_*α*_) corresponds to the repeatability of the random vectors.

The method calculates the average repeatability of a permuted random dataset from the sample. Repeatability calculation requires a statistic similar to a t-test

$$d_{\alpha }\;\left(g\right)=\frac{\left|\bar{x_{g}}-\bar{y_{g}}\right|}{\alpha_{1}+\alpha_{2}s_{g}},$$ (10)

where, $\bar{x_{g}}$ and $\bar{y_{g}}$ are the means of the genes *g* in the samples of groups *x* and *y* respectively, *S*_*g*_ is a standard error.

**4. Significant analysis of microarrays.** For two independent gene samples with normal distribution, the traditional t-test [39] can be represented as

$$t=\frac{\bar{g_{1}}-\bar{g_{2}}}{\sqrt{\frac{s_{g_{1}}^{2}}{n_{1}}+\frac{s_{g_{2}}^{2}}{n_{2}}}},$$ (11)

where, $s_{g_{1}}$ and $s_{g_{2}}$ are the variances of the gene expression *g*_1_ and *g*_2_ on different conditions. For genes with low expressed level, $s_{g_{1}}$ and $s_{g_{2}}$ are usually tiny, producing a large *t* from Eq (11), leading to a misjudgement. To overcome this shortcoming, Tusher et al., Smyth and Broberg proposed the methods significant analysis of microarrays (SAM), B-statistics, and samroc, respectively [11, 14, 23].

SAM employs a method that is similar to t-statistics and permutation test to estimate the false discovery rate [14], and mitigates the small variance problem involved in traditional t-test by adding a small positive constant *s*_0_. SAM statistic is defined as

$$t_{s}\approx \frac{\bar{g_{1}}-\bar{g_{2}}}{\sqrt{\frac{s_{g_{1}}^{2}}{n_{2}}+\frac{s_{g_{2}}^{2}}{n_{1}}}+s_{0}}.$$ (12)

#### 2.2.3 Description of the experiments

The experiments in this study were based on the platform with Windows 7, 32-bit operating system, i5-3470@3.2GHz CPU, 4 GBs memory. MIC was implemented by employed the function written in Matlab provided in work [40] (the core of the code is written in C), and the benchmarks were implemented by using R language functions provided by Bioconductor (V3.7). Except the parameter B (Bootstrap count) of ROTS, all the parameters of the functions were used the defaults. In addition, all experiments related to runtime were run in a single task (i.e., only the experimental program is running). The real data were only used to the experiment for obtaining the probability density distributions of gene expression profiles, while the simulation data were used to the other experiments.

## 3 Results

### 3.1 Probability density distributions of real data

**Fig 1:**
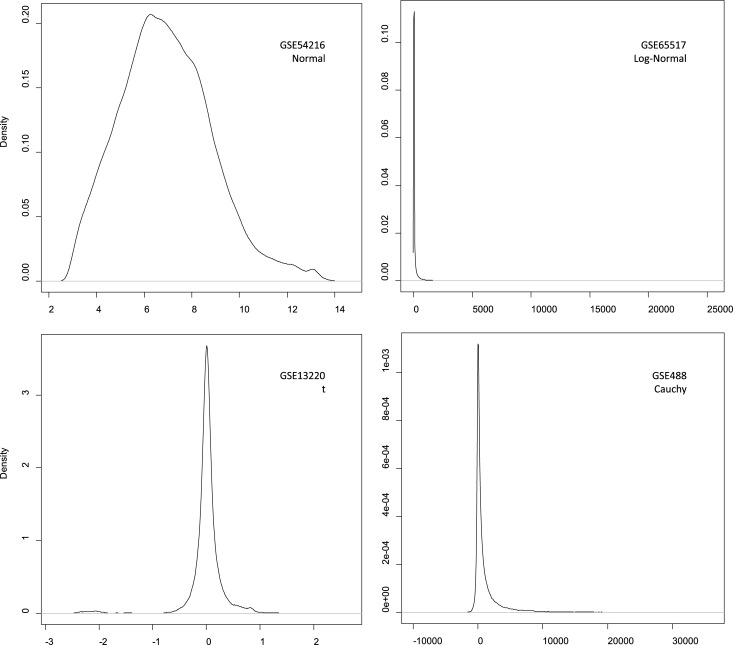
Four typical density distributions of real data.

We plotted the probability density curves of the 100 real datasets, and calculated their means and variances. Based on the shapes of the curves, the density distributions were firstly assumed by artificial ways. Next, we tested the accuracy of the assumption by the following process: (1) employ a function to generate a dataset with the assumed distribution, and adjust the function parameters so that the probability density curve of the data is close to the assumed distribution curve; (2) the mean and variance of the generated data are calculated and compared with the real values. Our experiments showed that although the density curve shape of the generated data with Weibull, gamma or chi-square distribution may be close to an assumed curve by suitable parameters, the mean and/or variance of the generated data are far from the real data. Finally, the experiment screened out four distributions as the probability density distributions of real data (Table 5). The typical density curves are shown in Fig 1, while all the 100 curves are shown in S1–S100 Figs.

**Table 5:** Probability density distributions of real data.

Distribution	Count
Log-normal	43
Normal	37
t	19
Cauchy	1

### 3.2 Test bootstrap count for ROTS

**Fig 2:**
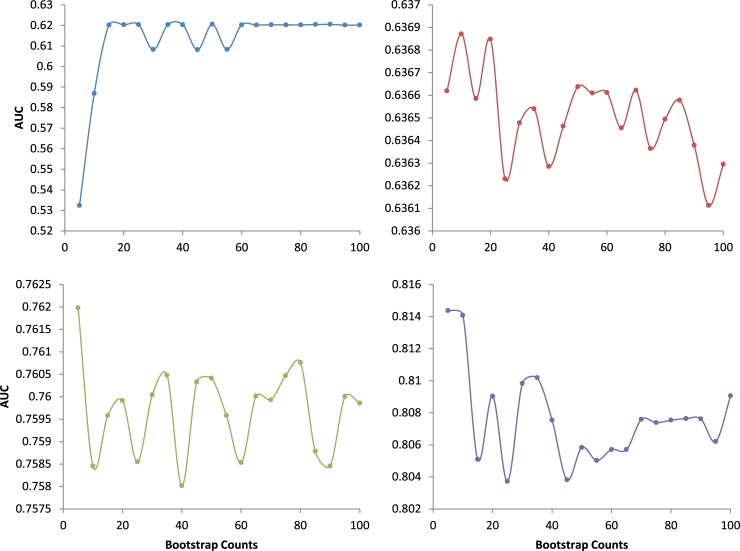
Bootstrap-AUC curves. The curve on upper-left, upper-right, lower-left and lower-right has a normal, log-normal, t or Cauchy distribution respectively. The Bootstrap counts of the 20 points are: 5, 10, 15, 20, 25, 30, 35, 40, 45, 50, 55, 60, 65, 70, 75, 80, 85, 90, 95, and 100, respectively.

ROTS uses Bootstrap sampling for statistical inference. The default Bootstrap count (parameter B) in the R function of ROTS is 1000. Our experiments showed that the ROTS runtime is proportional to B (see S101 Fig). At B = 1000, the runtime of a single dataset was 4.74 minutes. To reduce unnecessary cost in runtime, we let ROTS analyse the group 1 simulation dataset of the four distributions with several B values and calculated their average AUCs, respectively. The results shown in Fig 2 indicate that the average AUC with normal distribution is most affected by B, and the others are much less affected by B. When B>20, the average AUC with normal distribution hardly increases, while it decreases instead in log-normal distribution. Therefore, the parameter B = 20 was employed in the subsequent experiments of ROTS. It should be noted that B = 20 is not the optimal parameter of t and Cauchy distributions, but (1) it is suboptimal, (2) the average AUCs of the two distributions are little affected by B, and (3) the occurrence of the two distributions in real data are very low probabilities. Thus, the parameter B = 20 of ROTS has few effects on the results.

### 3.3 Performance evaluation based on noise-free data

**Fig 3:**
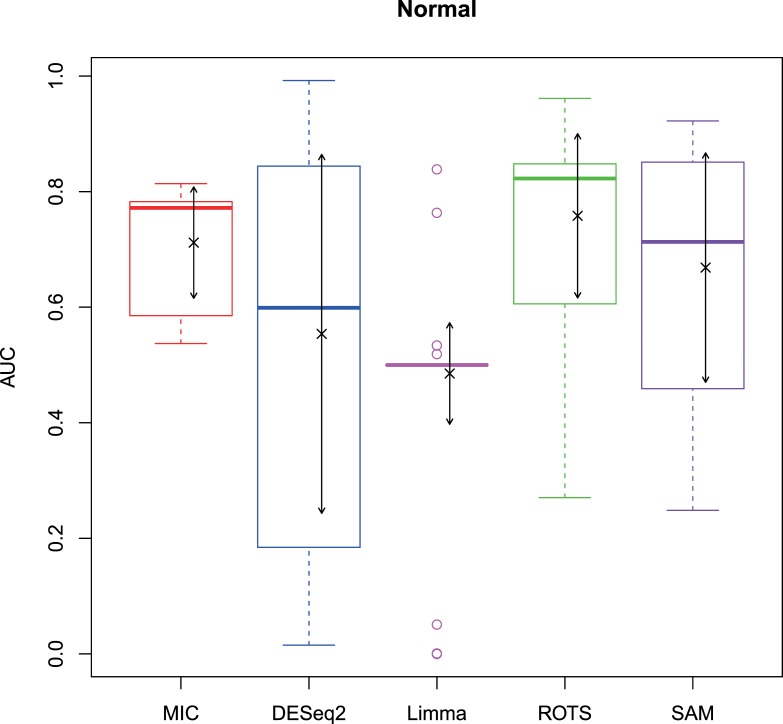
AUC boxplots on normal data ‘×’s are the means. Bidirectional arrows represent ±1σ.

**Fig 4:**
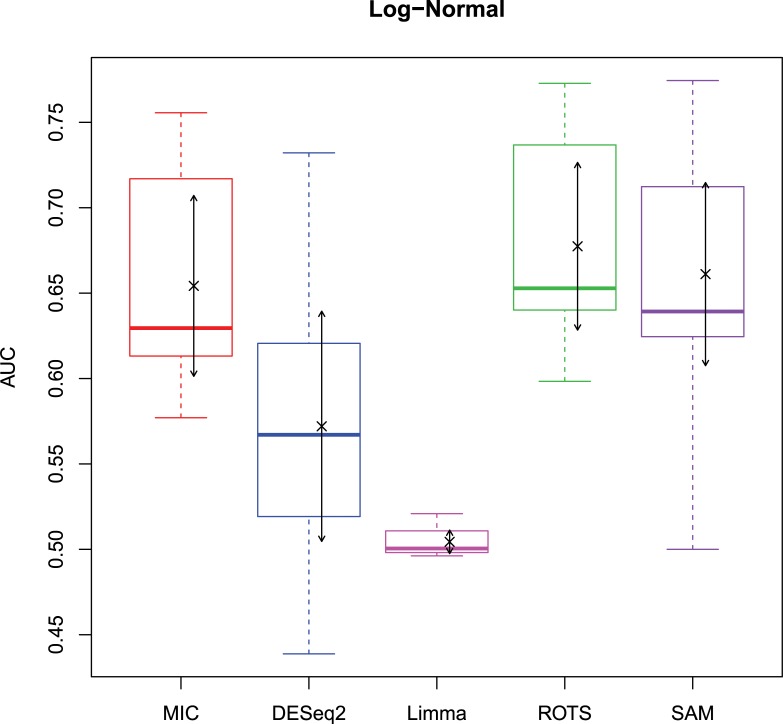
AUC boxplots on log-normal data ‘×’s are the means. Bidirectional arrows represent ±1σ.

**Fig 5:**
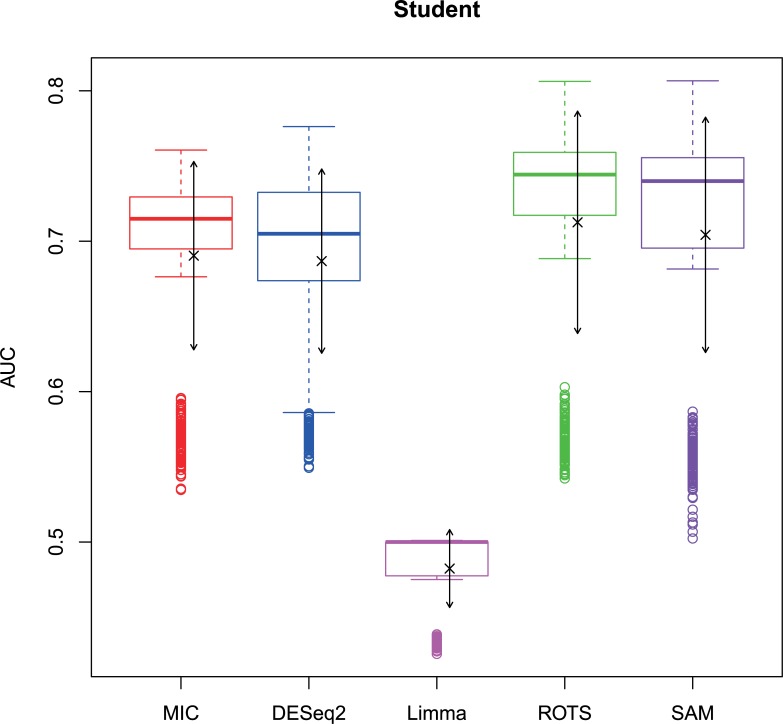
AUC boxplots on t data ‘×’s are the means. Bidirectional arrows represent ±1σ.

**Fig 6:**
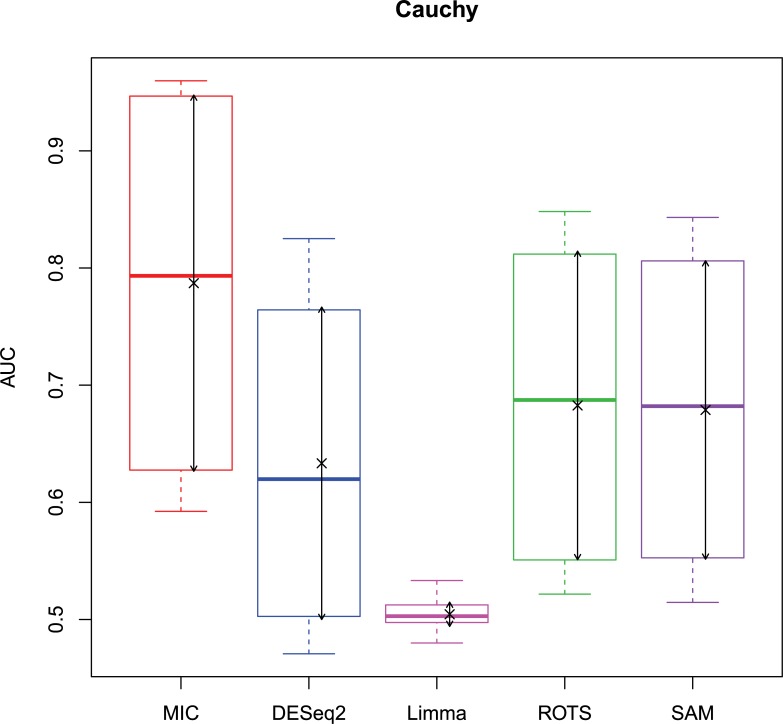
AUC boxplots on Cauchy data ‘×’s are the means. Bidirectional arrows represent ±1σ.

The 2,500 simulation datasets generated in Section 2.1.2 were analysed by MIC and its benchmarks. The ROC curves were plotted based on the analysis results, and the AUCs of the curves were calculated to characterize the abilities of identifying differentially expressed gene of the methods. Then, based on the AUCs, the boxplots were drawn, which are shown in Figs 3–6.

The identification of differentially expressed genes is a typical binary classification. For a binary-classification method, when its AUC is equal to 0.5, the prediction of the method is just a random guess and loses the predicting value; and the prediction is worse than a random guess when AUC < 0.5. Therefore, for testing the performance of MIC further, we counted the five methods on the four distributions when AUC ≤ 0.5, respectively (Table 6).

**Table 6:** Counts of AUC ≤ 0.5.

Distribution	MIC	DESeq2	Limma	ROTS	SAM
Normal	0	378	896	33	300
Log-Normal	0	141	438	0	1
t	0	0	490	0	0
Cauchy	0	44	73	0	0
Total	0	563	1897	33	301
Ratio (%)	0	22.52	75.88	1.32	12.04

Note: The counts come from the 2,500 simulation datasets, one for each.

### 3.4 Performance evaluation based on noisy data

**Fig 7:**
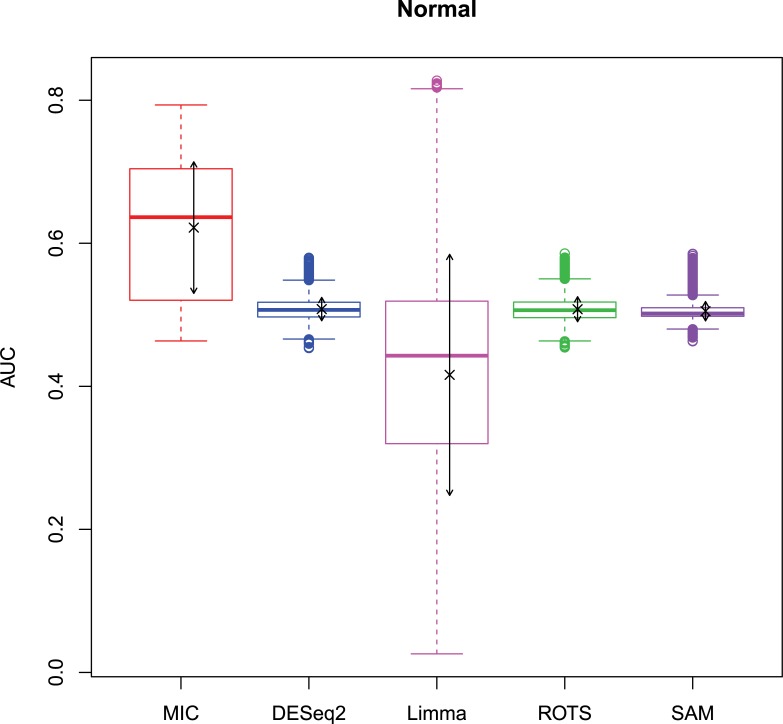
AUC boxplots on normal noisy data ‘×’s are the means. Bidirectional arrows represent ±1σ.

**Fig 8:**
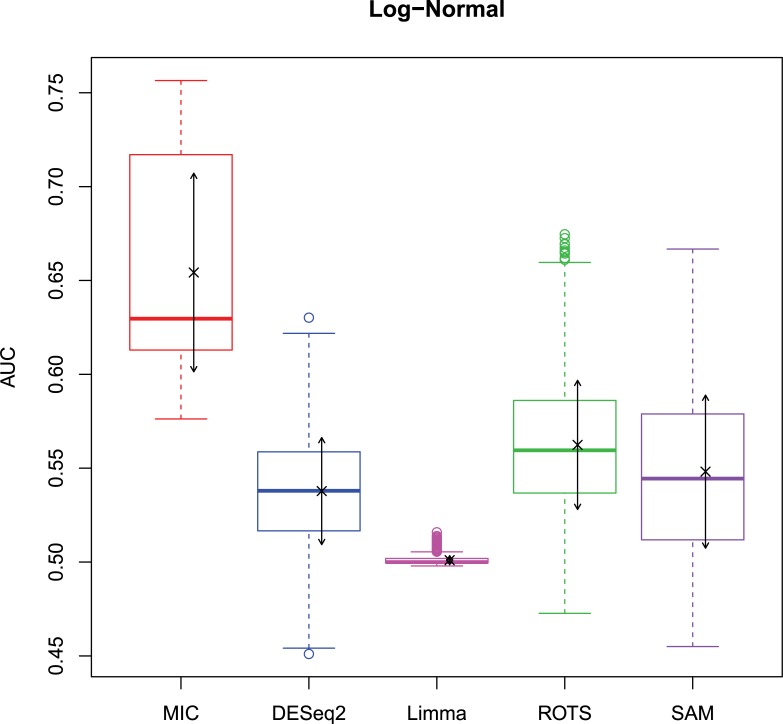
AUC boxplots on log-normal noisy data ‘×’s are the means. Bidirectional arrows represent ±1σ.

**Fig 9:**
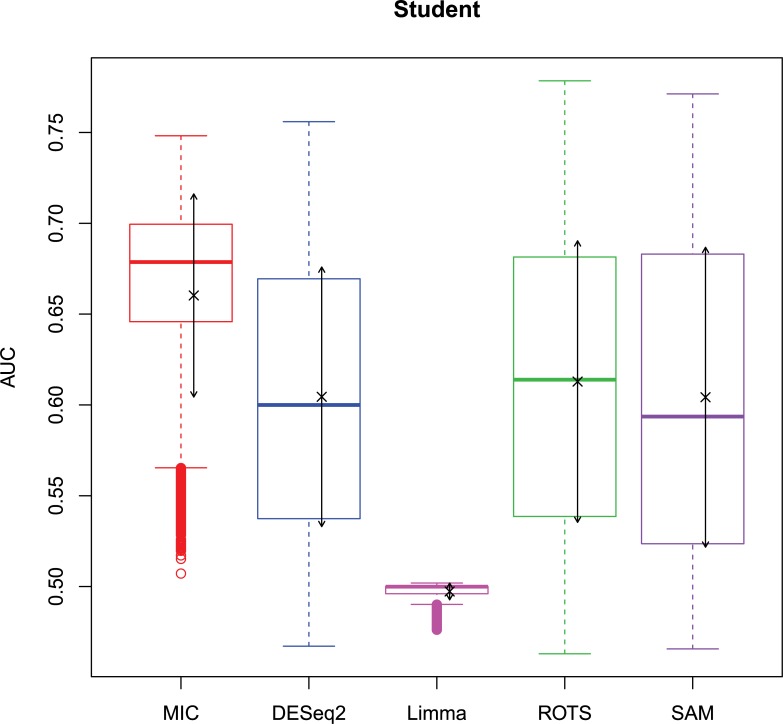
AUC boxplots on t noisy data ‘×’s are the means. Bidirectional arrows represent ±1σ.

**Fig 10:**
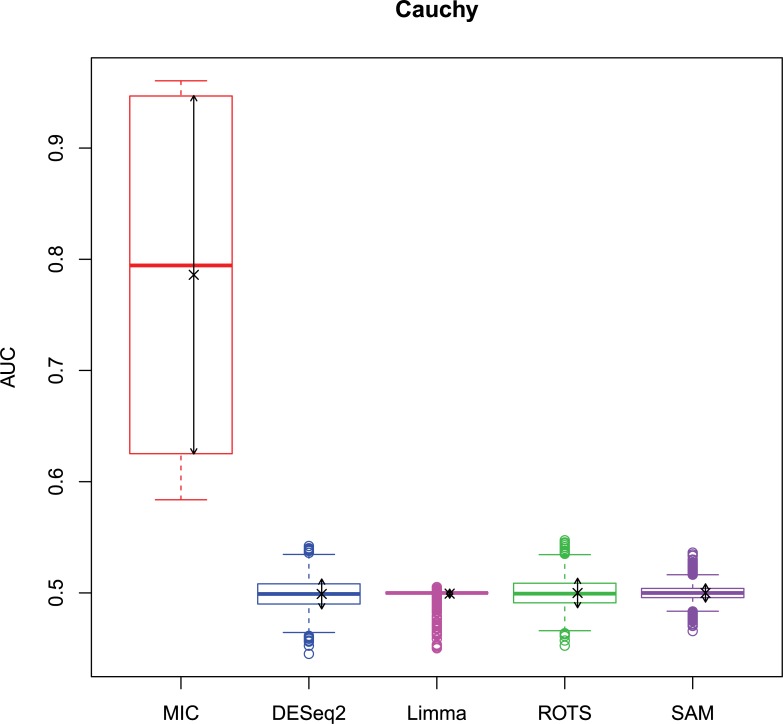
AUC boxplots on Cauchy noisy data ‘×’s are the means. Bidirectional arrows represent ±1σ.

A real gene expression profile is inevitably mixed with a great amount of noise [41], which may lead the identifying method to yielding numerous false positives. The noise immunity is one of the important performance indicators for a method used to identify differentially expressed genes. This study simulated noisy expression data by adding white noise to the simulation data generated in Section 2.1.2. The noise intensity in the noisy data is represented by the signal-to-noise ratio (SNR). And, the larger SNR is, the lower noise is. In our experiments, 11 kinds of white noise with different intensity levels were added to each dataset, where the 11 noisy levels are SNRs of 0~10 with step 1. Based on the results of the experiments, the boxplots of the methods on the distributions were produced on the model of Section 3.3, which are shown in Figs 7–10.

In order to explore the change of AUC with noise intensity, we also made linear fittings to the noise-AUC points. There are some errors naturally in the fitted lines affected by the errors of the methods. Thus, in our experiments, the fitted line was considered as a straight line approximately while the slope of the line is within the range of ±1.0×10^−4^. A horizontal noise-AUC fitted line indicates that the method producing the line is almost free from the noise; the line with a slope less than 0 represents the performance of the method is naturally affected by the noise intensity, and on the contrary, the method with a slope greater than 0 may be abnormal. Table 7 shows the counts of the noise-AUC fitted lines with a slope greater than 0, where the counts of the approximate horizontal straight line were removed. The fitted lines of all the methods on the four distributions are shown in S102–S121 Figs.

**Table 7:** Counts of fitted lines with a slope greater than 0.

Distribution	MIC	DESeq2	Limma	ROTS	SAM
Normal	2	79	823	88	56
Log-Normal	0	38	0	0	8
t	0	0	100	3	3
Cauchy	0	47	12	50	24
Total	2	164	935	141	91
Ratio (%)	0.08	6.56	37.40	5.64	3.64

Note: The counts come from the 2,500 simulation datasets, one for each. And, the approximately horizontal lines have been removed.

### 3.5 Algorithm runtimes test

Although the algorithm runtime test could not accurately reflect the difference in speed performance since the implementations of the methods are different, it is still possible to tell a summary distinction. Here, the five methods were employed to analyse the first simulation dataset in the first group of each distribution, respectively. The runtimes of the methods were recorded, which are shown in Table 8.

**Table 8:** Algorithm runtimes (unit: second).

Distribution	Method				
	MIC	DESeq2	Limma	ROTS	SAM
Normal	0.72	6.37	0.30	9.59	1.06
Log-Normal	0.60	6.39	0.41	9.08	1.46
Student	0.89	5.36	0.28	9.08	1.06
Cauchy	0.71	9.00	0.34	9.06	1.13
Total	2.92	27.11	1.34	36.81	4.71

## 4 Discussions

Identification of differentially expressed genes is a binary classification problem in data mining. To improve the performance of a binary classification method for expressed gene profiles further, we constructed the model (3), where the sample phenotype *T* is the dependent variable and gene *j* (*g*_*j*_) is the independent variable. Based on this model, differentially expressed genes can be screened by simply calculating the MIC values of all genes in the expression profile. The calculation does not involve any parameter assumptions or estimations.

### 4.1 Probability density distributions of real gene expression profiles

By analysing 100 real expression datasets, it was found that the normal and log-normal distributions account for up to 80% (37 normal distributions and 43 log-normal distributions, see Table 5). Since the normal distribution and the log-normal distribution can be easily converted to each other, it is feasible to assume that a gene expression profile is normal distribution in existing studies. In addition to the two distributions, there are t distribution of 19% and Cauchy distribution of 1%, indicating that besides the normal distribution, the t and Cauchy distribution (at least the t distribution) need to be considered in a comprehensive study of gene expression. Moreover, although the distributions of Weibull, gamma and chi-square are also possible based on the density curves shape, it was found that either forms or the means and variances of the curves simulated by the three distributions are far from the real data. Thus, the three distributions are not likely to appear in the density distributions of real gene expression profiles.

### 4.2 Performance of identifying DEGs by MIC on noise-free data

Since the Bootstrap used in ROTS will cost a lot of runtime, we tested the optimal Bootstrap count (i.e., the parameter B of ROTS) for the method. Our experiments showed that B = 20 is the best compromise case between the accuracy and runtime of ROTS for identifying differentially expressed genes, and the runtime has a good linear relationship with the B. All experiments on ROTS were done based on this parameter.

The study used AUC as a characterization of the ability of identifying differentially expressed genes for each method. The boxplots of the four distributions in Figs 3–6 show that the identifying ability of MIC is significantly stronger than Limma and DESeq2 methods (Limma is also significantly weaker than the other benchmarks). The identifying ability of MIC in the normal distribution data is second only to that of ROTS (the AUC median is 6.19% smaller), ranked no. 2; the AUC median in the log-normal distribution is slightly smaller than that of ROTS and SAM (are smaller 3.57% and 1.52% respectively), ranked no. 3, and that in t distribution is also slightly smaller than ROTS and SAM (are smaller 3.95% and 3.39% respectively), ranked no. 3 too, while that in Cauchy distribution is significantly better than the four benchmarks. In addition, an AUC variance can reflect the adaptability of a method to the data. A smaller AUC variance means that the method is more adaptable to the data, that is, the method does not bring large fluctuations in its result caused by the overall change in expressed levels. Figs 3–6 show that Limma has the smallest variance, and MIC is better than or equivalent to the other three methods. Among the four distributions, any of methods has a distribution that its variance is weaker than the other distributions (MIC, ROTS and SAM are Cauchy, DESeq2 and Limma are Normal). So far, we can conclude that the performance of identifying differentially expressed genes by MIC on noise-free data is significantly better than that of Limma and DESeq2, while it is almost same with ROTS and SAM. MIC is weaker than Limma in terms of adaptability to changes in expressed levels, and is almost same with DESeq2, ROTS, and SAM. The adaptabilities of all the methods to distributions are similar. However, we could consider that the adaptabilities of MIC, ROTS and SAM are better than the other two methods, because the possibility of density distribution of Cauchy in real data is significantly lower than a normal distribution.

In addition, for a binary classifier, when AUC = 0.5, the method has no practical value, while AUC<0.5 indicates that the method has serious defects. Fewer AUC ≤ 0.5 indicates that the method is more robust and adaptable to data. In the results of AUC ≤ 0.5 shown in Table 6, MIC does not exhibit the case of AUC ≤ 0.5, which is significantly better than the benchmarks.

Therefore, compared to the existing methods, MIC is in the first tier in the performance of identifying differentially expressed genes, and it has stronger robust and higher data adaptability.

### 4.3 Noise immunity of MIC in identifying differentially expressed genes

The noise in a gene expression profile is an important factor affecting the accuracy of an identifying method, especially for the genes with low expressed levels. In order to investigate the noise immunity of MIC, we tested the identifying performance of MIC in a noisy environment by adding white noise to a noise-free dataset. Our experiments used SNR to represent the noise intensity in the data. The results (see Figs 7–10) show that the AUC medians of MIC in the noisy data is significantly better than the benchmarks, while the overall variance is weaker than the benchmarks. However, the change to the variance of MIC among the four distributions is greatly smaller than the benchmarks, and the variances of MIC on noisy data are similar to that on noise-free data. It means that the noise immunity of MIC is significantly better than the benchmarks.

To further investigate the relationship between performance and noise intensity for a method, we made linear fitting for the (1-SNR)-AUC scatter points. If a method has excellent noise immunity, its fitted line should be approximately horizontal, and there are no (or almost no) cases where the slopes of the lines are greater than 0. Table 7, the counts of the fitted line with a slope greater than 0, show that the count of MIC is only 2, which is strikingly better than the benchmarks. S102–S121 Figs also show that MIC is the only one of the methods has no (1-SNR)-AUC (where AUC is a mean) fitted line with a slope greater than zero in all distributions.

Thus, compared to the existing methods, the noise immunity of MIC shows an obvious advantage.

### 4.4 Comparison of algorithm runtimes

Since the implementations of the methods are different, the runtime comparison can only be rough. The runtimes shown in Table 8 indicate that the runtime of MIC is greatly longer than Limma, slightly shorter than SAM, but significantly shorter than DESeq2 and ROTS. It shows that MIC is overall faster than the most existing methods. The reason why Limma's runtime is the shortest among all methods is mainly because it assumes that the variables are linear relationship, which makes the computational complexity significantly smaller than the other methods.

### 4.5 Advantages and disadvantages of MIC

MIC is a non-parametric statistical method with good noise immunity. It has better ability to discover non-functional relations than the existing methods in exploring bivariate relations. Furthermore, it has a good uniformity to function relations [29] (i.e., MIC can yield almost the same value for any function relations). A gene expression profile has usually a lot of noise [41] and the function relation between the phenotype and gene expressed levels is not clear, thus, MIC is very suitable for analysis of gene expression data.

The deficiencies of MIC are mainly reflected in the fact that it rasterizes (i.e., discretizes) the continuous gene expression data, which leads it to be an approximation method and reduce its accuracy.

## 5 Conclusion

In summary, the result of the analysis of the real expression profiles suggested that the probability density distribution of a gene expression data may be normal, log-normal, t or Cauchy, and is mostly normal or log-normal distribution (accounting for 80%). Due to the ease of conversion between normal and log-normal distributions, we could assume the density distribution of a gene expression profile is normal in a simple analysis. However, for more accurate analysis, at least a t-distribution (accounting for 19% in the real data) is needed besides a normal. In addition, the simulation experiments reveal that MIC is not weaker than the existing methods (in the top tier) in the performance of identifying differentially expressed genes, and it is superior to existing methods in adaptability and noise immunity (especially its noise immunity). And, MIC has a shorter runtime. In conclusion, MIC has a good performance of identifying differentially expressed genes, noise immunity and a shorter runtime. It is an excellent method for identifying differentially expressed genes.

## Supporting information

S1 Fig Density of GSE26585. (EPS)

S2 Fig Density of GSE488. (EPS)

S3 Fig Density of GSE100642. (EPS)

S4 Fig Density of GSE10072. (EPS)

S5 Fig Density of GSE103184. (EPS)

S6 Fig Density of GSE103430. (EPS)

S7 Fig Density of GSE106635. (EPS)

S8 Fig Density of GSE106912. (EPS)

S9 Fig Density of GSE110398. (EPS)

S10 Fig Density of GSE12196. (EPS)

S11 Fig Density of GSE12452. (EPS)

S12 Fig Density of GSE13220. (EPS)

S13 Fig Density of GSE13597. (EPS)

S14 Fig Density of GSE13911. (EPS)

S15 Fig Density of GSE14304. (EPS)

S16 Fig Density of GSE16765. (EPS)

S17 Fig Density of GSE18608. (EPS)

S18 Fig Density of GSE20347. (EPS)

S19 Fig Density of GSE20466. (EPS)

S20 Fig Density of GSE20489. (EPS)

S21 Fig Density of GSE20586. (EPS)

S22 Fig Density of GSE21947. (EPS)

S23 Fig Density of GSE22356. (EPS)

S24 Fig Density of GSE22671. (EPS)

S25 Fig Density of GSE23400. (EPS)

S26 Fig Density of GSE24342. (EPS)

S27 Fig Density of GSE24988. (EPS)

S28 Fig Density of GSE25156. (EPS)

S29 Fig Density of GSE26623. (EPS)

S30 Fig Density of GSE2685. (EPS)

S31 Fig Density of GSE27114. (EPS)

S32 Fig Density of GSE29110. (EPS)

S33 Fig Density of GSE29633. (EPS)

S34 Fig Density of GSE3017. (EPS)

S35 Fig Density of GSE30502. (EPS)

S36 Fig Density of GSE31564. (EPS)

S37 Fig Density of GSE31738. (EPS)

S38 Fig Density of GSE32515. (EPS)

S39 Fig Density of GSE3268. (EPS)

S40 Fig Density of GSE33003. (EPS)

S41 Fig Density of GSE33373. (EPS)

S42 Fig Density of GSE33459. (EPS)

S43 Fig Density of GSE33463. (EPS)

S44 Fig Density of GSE33672. (EPS)

S45 Fig Density of GSE34400. (EPS)

S46 Fig Density of GSE34667. (EPS)

S47 Fig Density of GSE34872. (EPS)

S48 Fig Density of GSE3494. (EPS)

S49 Fig Density of GSE3519. (EPS)

S50 Fig Density of GSE35240. (EPS)

S51 Fig Density of GSE37404. (EPS)

S52 Fig Density of GSE37902. (EPS)

S53 Fig Density of GSE38531. (EPS)

S54 Fig Density of GSE38783. (EPS)

S55 Fig Density of GSE39549. (EPS)

S56 Fig Density of GSE41221. (EPS)

S57 Fig Density of GSE46727. (EPS)

S58 Fig Density of GSE46728. (EPS)

S59 Fig Density of GSE47406. (EPS)

S60 Fig Density of GSE48964. (EPS)

S61 Fig Density of GSE49382. (EPS)

S62 Fig Density of GSE49486. (EPS)

S63 Fig Density of GSE50604. (EPS)

S64 Fig Density of GSE5281. (EPS)

S65 Fig Density of GSE53122. (EPS)

S66 Fig Density of GSE54129. (EPS)

S67 Fig Density of GSE54216. (EPS)

S68 Fig Density of GSE54350. (EPS)

S69 Fig Density of GSE54917. (EPS)

S70 Fig Density of GSE55503. (EPS)

S71 Fig Density of GSE57002. (EPS)

S72 Fig Density of GSE5859. (EPS)

S73 Fig Density of GSE61140. (EPS)

S74 Fig Density of GSE62598. (EPS)

S75 Fig Density of GSE6414. (EPS)

S76 Fig Density of GSE64670. (EPS)

S77 Fig Density of GSE64718. (EPS)

S78 Fig Density of GSE65517. (EPS)

S79 Fig Density of GSE6720. (EPS)

S80 Fig Density of GSE67376. (EPS)

S81 Fig Density of GSE67492. (EPS)

S82 Fig Density of GSE67865. (EPS)

S83 Fig Density of GSE68918. (EPS)

S84 Fig Density of GSE7124. (EPS)

S85 Fig Density of GSE71868. (EPS)

S86 Fig Density of GSE7197. (EPS)

S87 Fig Density of GSE75037. (EPS)

S88 Fig Density of GSE7511. (EPS)

S89 Fig Density of GSE7567. (EPS)

S90 Fig Density of GSE7592. (EPS)

S91 Fig Density of GSE7670. (EPS)

S92 Fig Density of GSE7881. (EPS)

S93 Fig Density of GSE79973. (EPS)

S94 Fig Density of GSE83077. (EPS)

S95 Fig Density of GSE8498. (EPS)

S96 Fig Density of GSE9687. (EPS)

S97 Fig Density of GSE9820. (EPS)

S98 Fig Density of GSE98634. (EPS)

S99 Fig Density of GSE99295. (EPS)

S100 Fig Density of GSE48200. (EPS)

S101 Fig Bootstraps-Elapse of ROTS. (TIF)

S102 Fig Average fitted line on Normal for MIC. (TIF)

S103 Fig Average fitted line on Normal for DESeq2. (TIF)

S104 Fig Average fitted line on Normal for Limma. (TIF)

S105 Fig Average fitted line on Normal for ROTS. (TIF)

S106 Fig Average fitted line on Normal for SAM. (TIF)

S107 Fig Average fitted line on Log-Normal for MIC. (TIF)

S108 Fig Average fitted line on Log-Normal for DESeq2. (TIF)

S109 Fig Average fitted line on Log-Normal for Limma. (TIF)

S110 Fig Average fitted line on Log-Normal for ROTS. (TIF)

S111 Fig Average fitted line on Log-Normal for SAM. (TIF)

S112 Fig Average fitted line on Student for MIC. (TIF)

S113 Fig Average fitted line on Student for DESeq2. (TIF)

S114 Fig Average fitted line on Student for Limma. (TIF)

S115 Fig Average fitted line on Student for ROTS. (TIF)

S116 Fig Average fitted line on Student for SAM. (TIF)

S117 Fig Average fitted line on Cauchy for MIC. (TIF)

S118 Fig Average fitted line on Cauchy for DESeq2. (TIF)

S119 Fig Average fitted line on Cauchy for Limma. (TIF)

S120 Fig Average fitted line on Cauchy for ROTS. (TIF)

S121 Fig Average fitted line on Cauchy for SAM. (TIF)

S1 File GEO Accession Numbers.pdf. (PDF)
