# Supplementary figures and images for: Density distribution of gene expression profiles and evaluation of using maximal information coefficient to identify differentially expressed genes
Source: PLoS One. 2019 Jul 17;14(7):e0219551. doi: 10.1371/journal.pone.0219551 (PMC6636747; doi:10.1371/journal.pone.0219551)

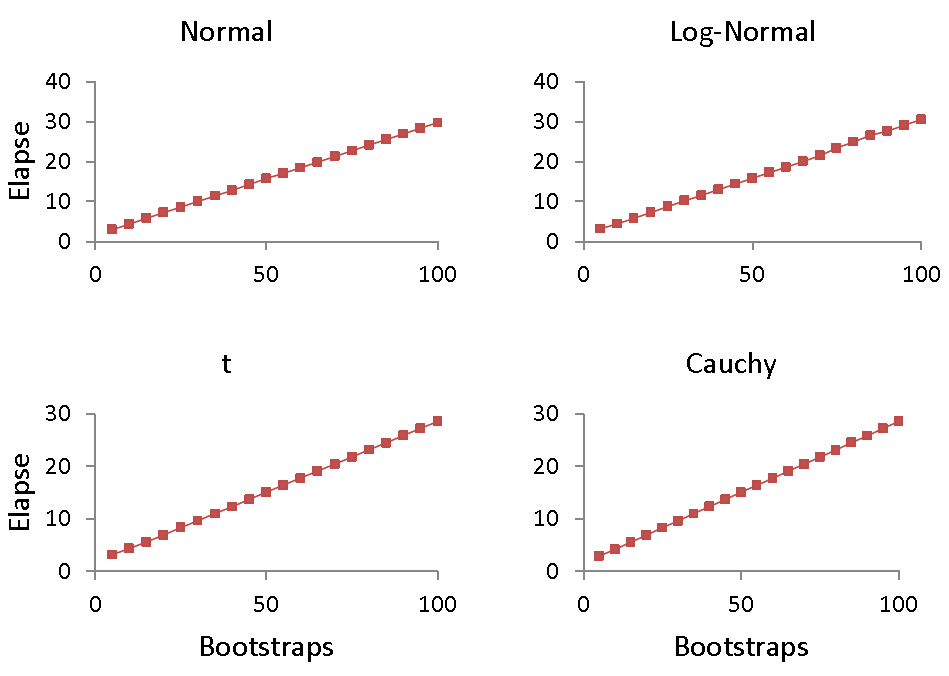

Supplement: S101 Fig — (TIF) [file pone.0219551.s101.tif]

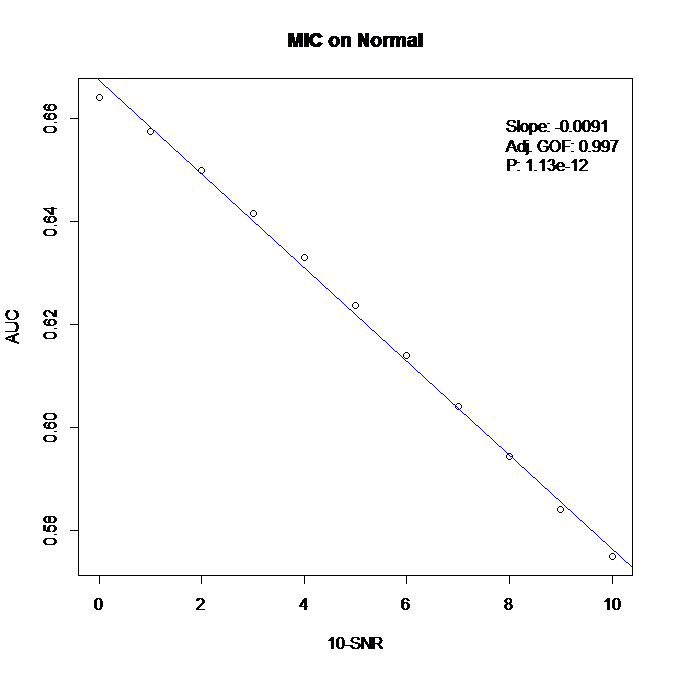

Supplement: S102 Fig — (TIF) [file pone.0219551.s102.tif]

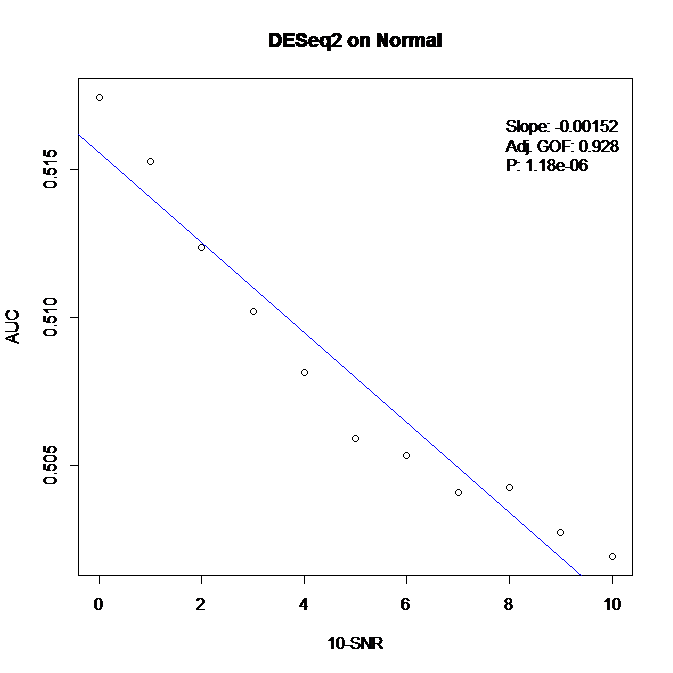

Supplement: S103 Fig — (TIF) [file pone.0219551.s103.tif]

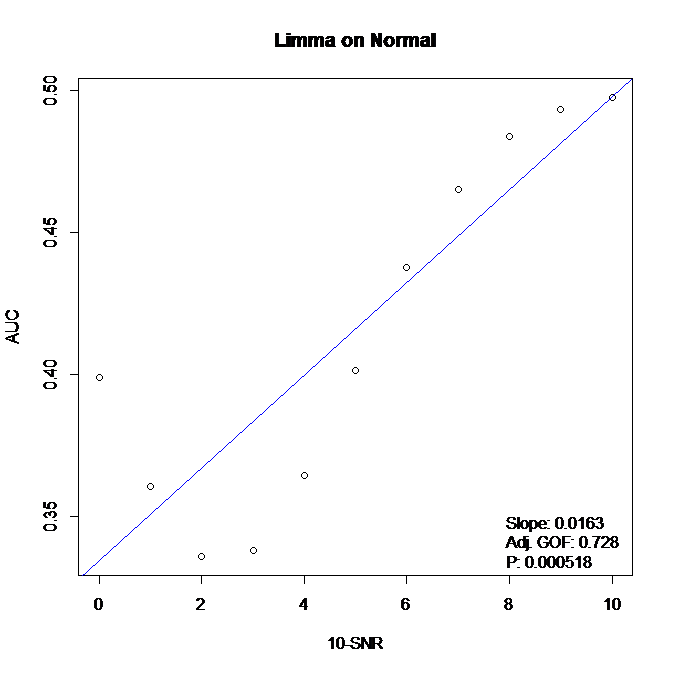

Supplement: S104 Fig — (TIF) [file pone.0219551.s104.tif]

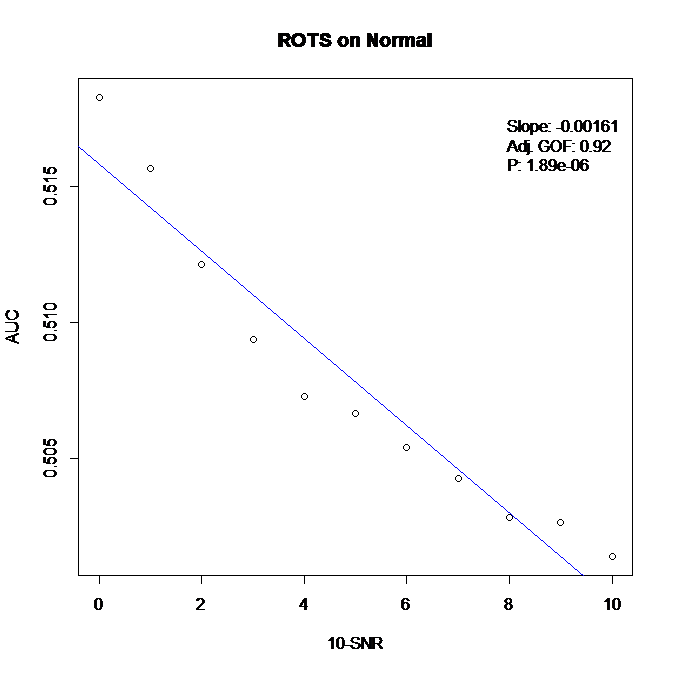

Supplement: S105 Fig — (TIF) [file pone.0219551.s105.tif]

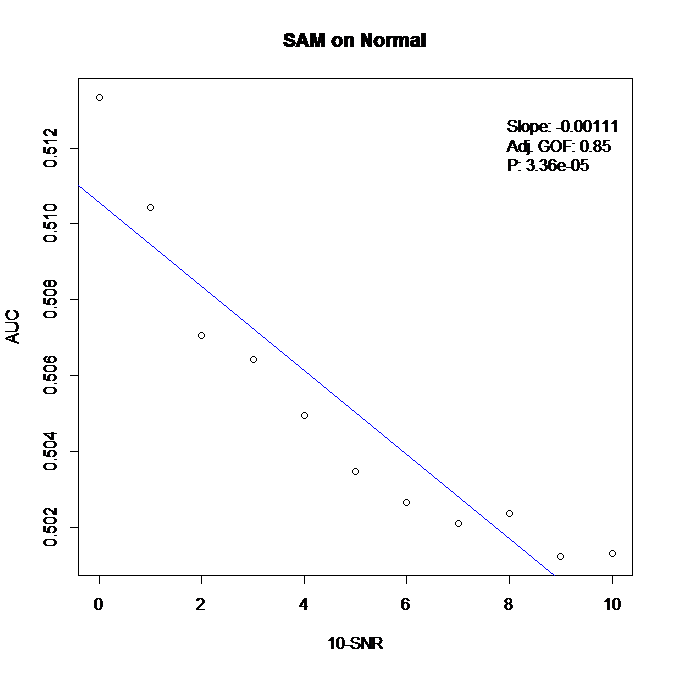

Supplement: S106 Fig — (TIF) [file pone.0219551.s106.tif]

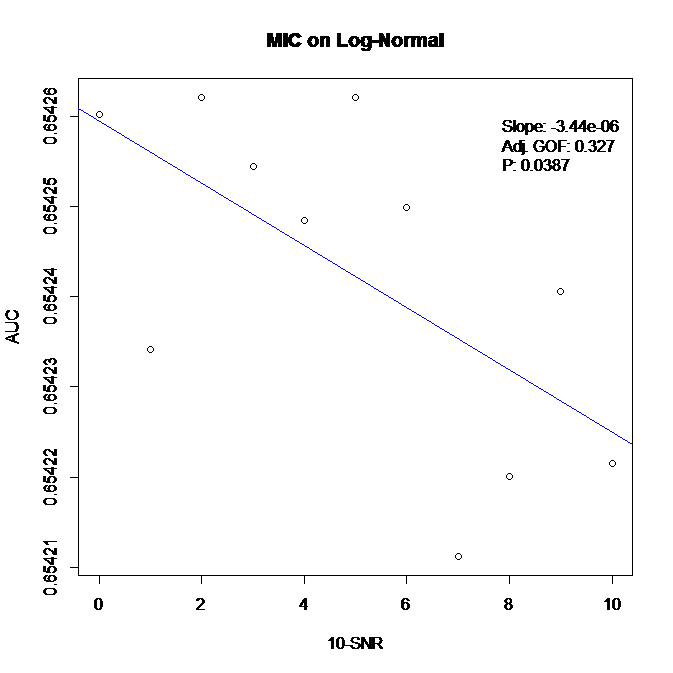

Supplement: S107 Fig — (TIF) [file pone.0219551.s107.tif]

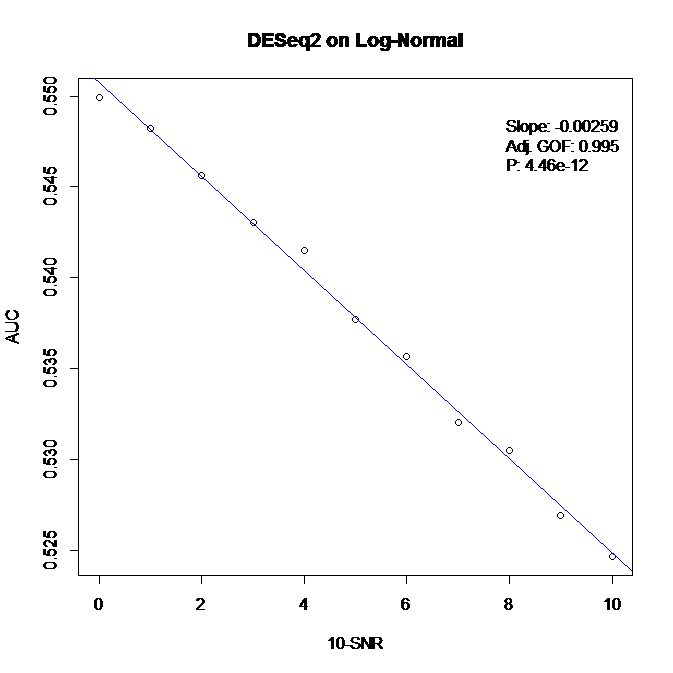

Supplement: S108 Fig — (TIF) [file pone.0219551.s108.tif]

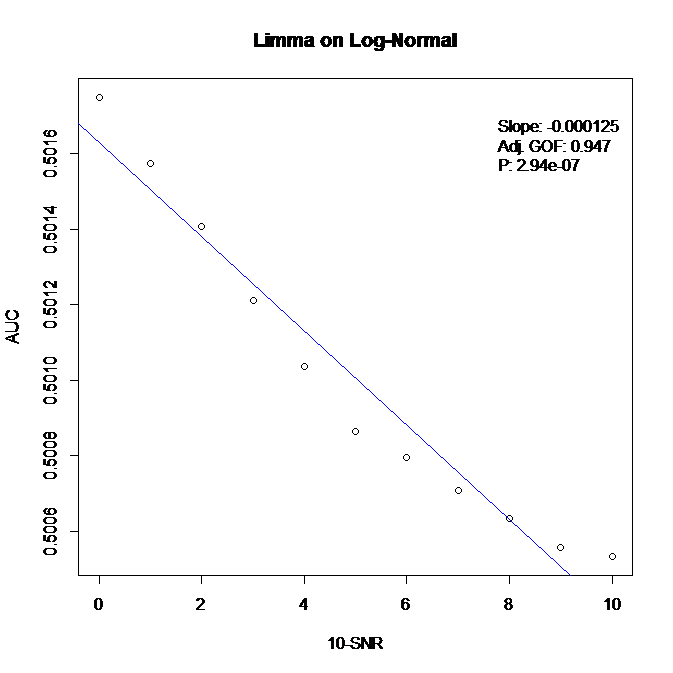

Supplement: S109 Fig — (TIF) [file pone.0219551.s109.tif]

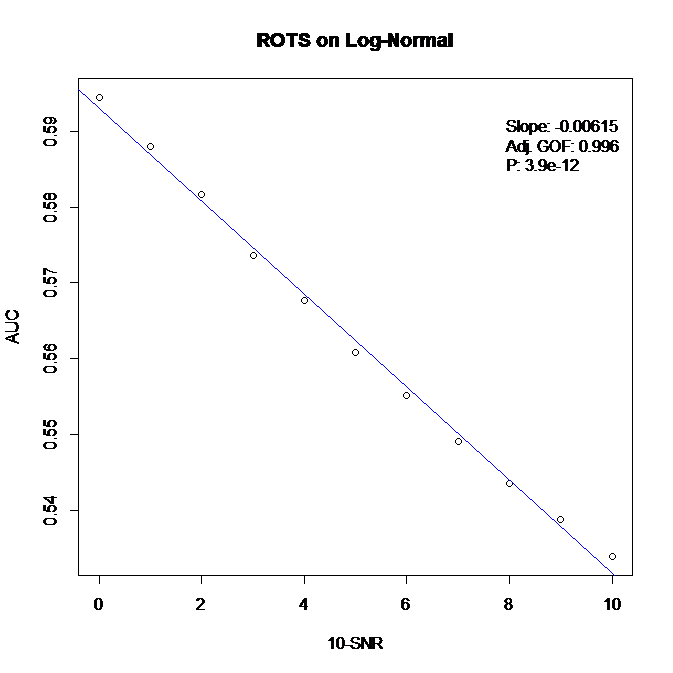

Supplement: S110 Fig — (TIF) [file pone.0219551.s110.tif]

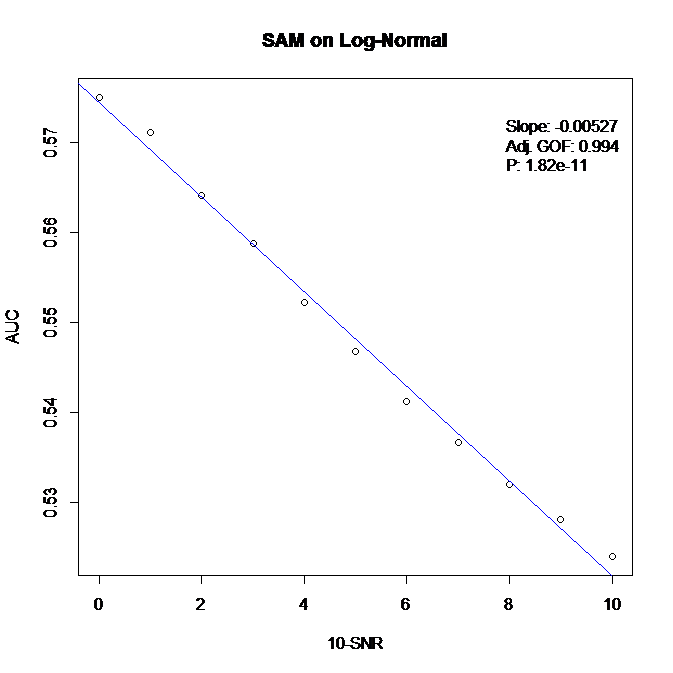

Supplement: S111 Fig — (TIF) [file pone.0219551.s111.tif]

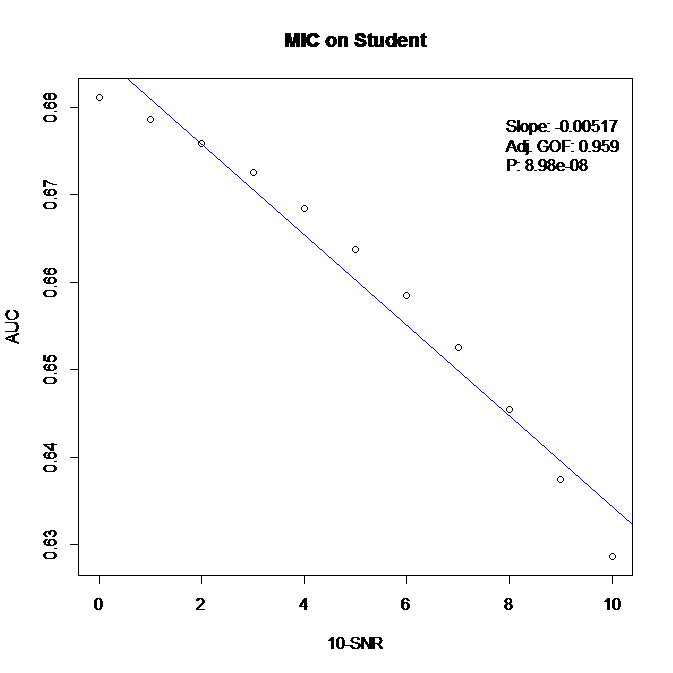

Supplement: S112 Fig — (TIF) [file pone.0219551.s112.tif]

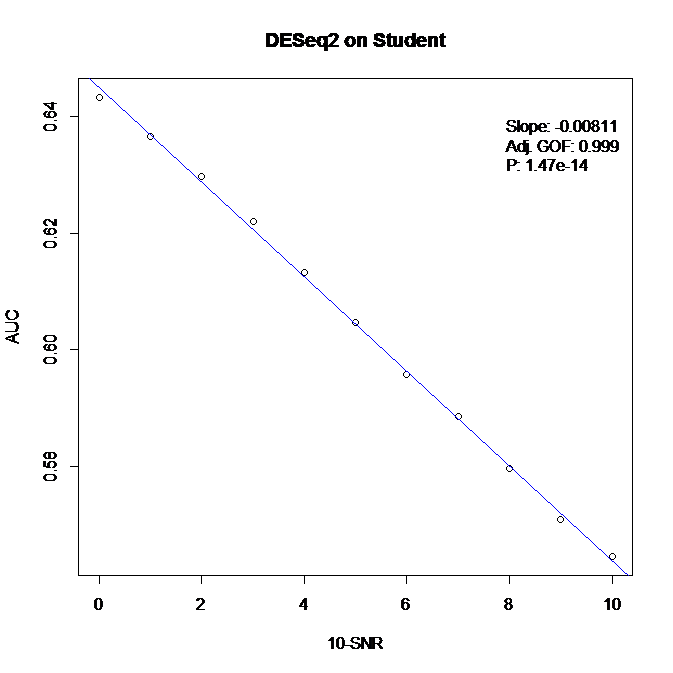

Supplement: S113 Fig — (TIF) [file pone.0219551.s113.tif]

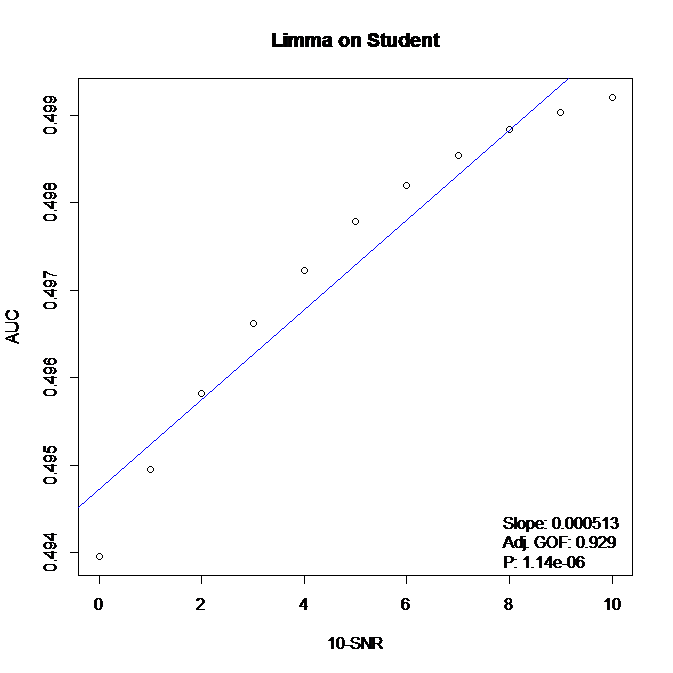

Supplement: S114 Fig — (TIF) [file pone.0219551.s114.tif]

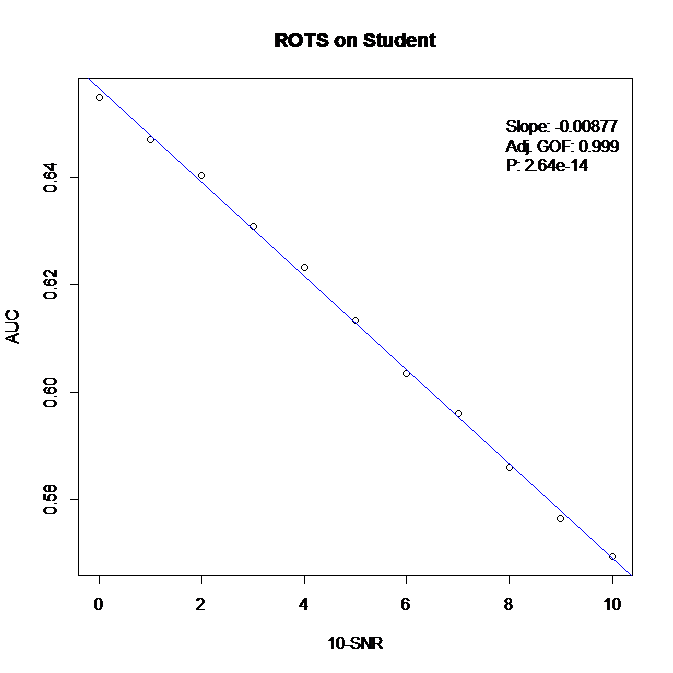

Supplement: S115 Fig — (TIF) [file pone.0219551.s115.tif]

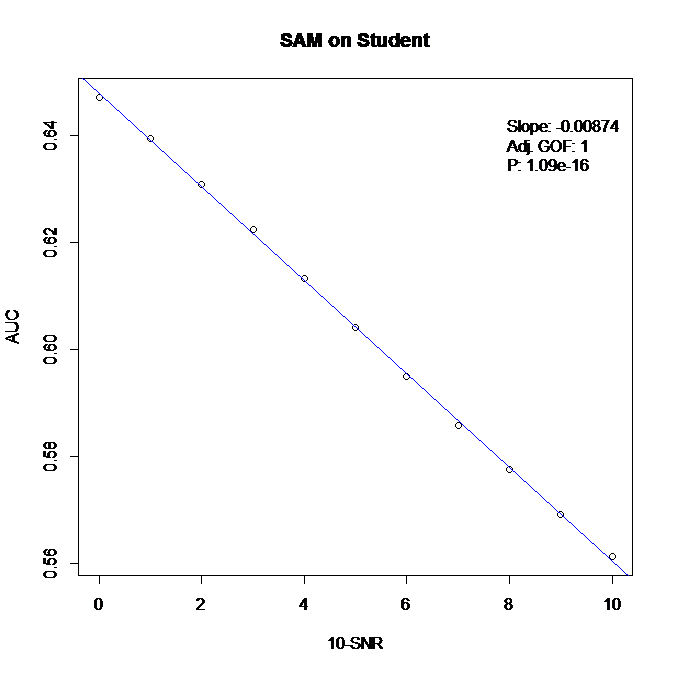

Supplement: S116 Fig — (TIF) [file pone.0219551.s116.tif]

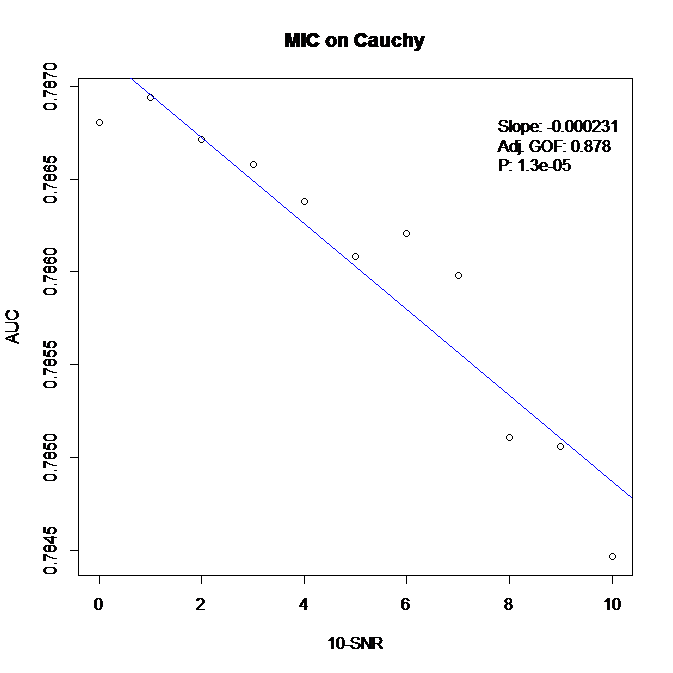

Supplement: S117 Fig — (TIF) [file pone.0219551.s117.tif]

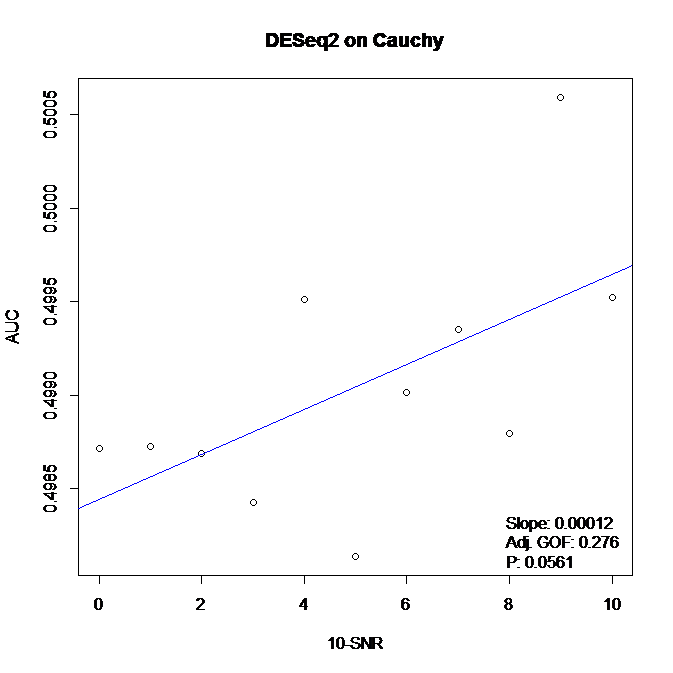

Supplement: S118 Fig — (TIF) [file pone.0219551.s118.tif]

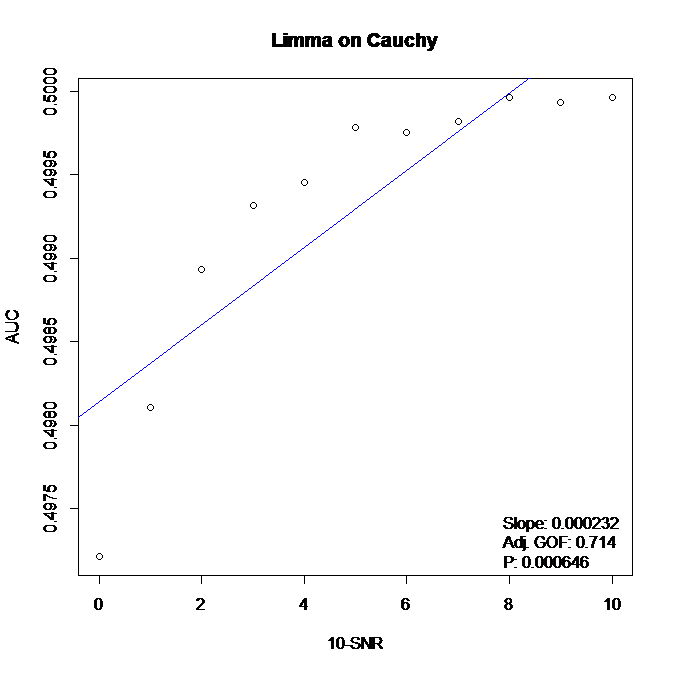

Supplement: S119 Fig — (TIF) [file pone.0219551.s119.tif]

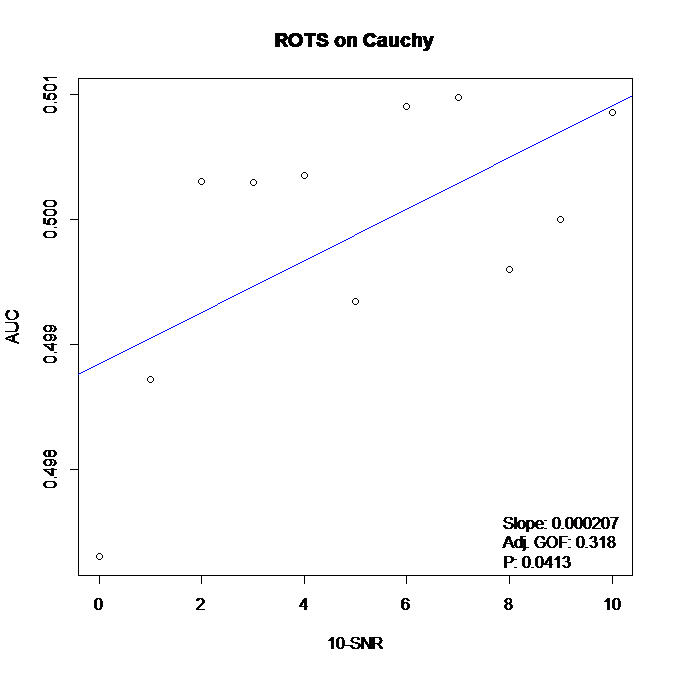

Supplement: S120 Fig — (TIF) [file pone.0219551.s120.tif]

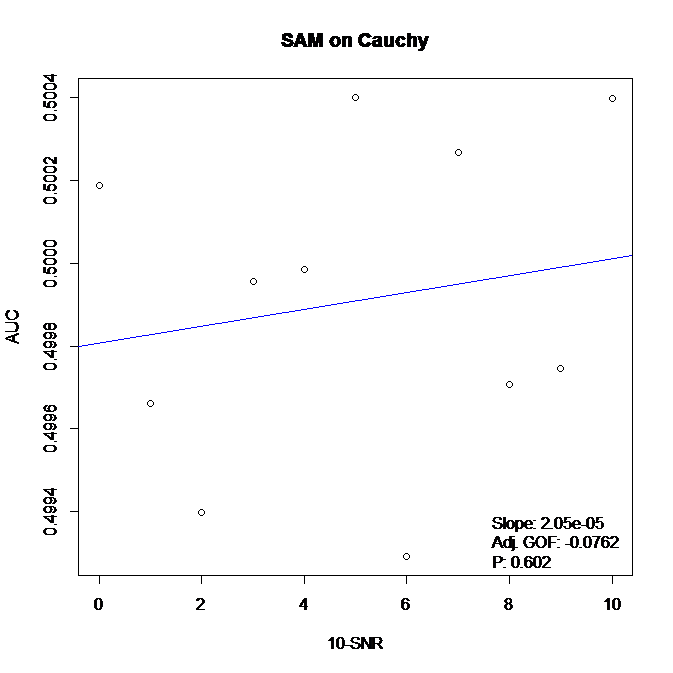

Supplement: S121 Fig — (TIF) [file pone.0219551.s121.tif]
