# Supplementary material for: Density distribution of gene expression profiles and evaluation of using maximal information coefficient to identify differentially expressed genes
Source: PLoS One. 2019 Jul 17;14(7):e0219551. doi: 10.1371/journal.pone.0219551 (PMC6636747; doi:10.1371/journal.pone.0219551)
Supplement: S1 File — (PDF) [file pone.0219551.s122.pdf]

All real gene expression profiles are available from the GEO database on NCBI. The accession numbers associated with the gene expression profiles used in this study are as bellow:

GSE26585	GSE488	GSE100642	GSE10072	GSE103184
GSE103430	GSE106635	GSE106912	GSE110398	GSE12196
GSE12452	GSE13220	GSE13597	GSE13911	GSE14304
GSE16765	GSE18608	GSE20347	GSE20466	GSE20489
GSE20586	GSE21947	GSE22356	GSE22671	GSE23400
GSE24342	GSE24988	GSE25156	GSE26623	GSE2685
GSE27114	GSE29110	GSE29633	GSE3017	GSE30502
GSE31564	GSE31738	GSE32515	GSE3268	GSE33003
GSE33373	GSE33459	GSE33463	GSE33672	GSE34400
GSE34667	GSE34872	GSE3494	GSE3519	GSE35240
GSE37404	GSE37902	GSE38531	GSE38783	GSE39549
GSE41221	GSE46727	GSE46728	GSE47406	GSE48964
GSE49382	GSE49486	GSE50604	GSE5281	GSE53122
GSE54129	GSE54216	GSE54350	GSE54917	GSE55503
GSE57002	GSE5859	GSE61140	GSE62598	GSE6414
GSE64670	GSE64718	GSE65517	GSE6720	GSE67376
GSE67492	GSE67865	GSE68918	GSE7124	GSE71868
GSE7197	GSE75037	GSE7511	GSE7567	GSE7592
GSE7670	GSE7881	GSE79973	GSE83077	GSE8498
GSE9687	GSE9820	GSE98634	GSE99295	GSE48200
